# Influence of Soil Nutrient Toxicity and Deficiency from Three Ecuadorian Climatic Regions on the Variation of Biological, Metabolic, and Nutritional Properties of *Moringa oleifera* Lam.

**DOI:** 10.3390/toxics10110661

**Published:** 2022-11-03

**Authors:** Raluca A. Mihai, Osmar S. Acurio Criollo, Jean P. Quishpe Nasimba, Erly J. Melo Heras, Dayana K. Galván Acaro, Pablo A. Landazuri Abarca, Larisa I. Florescu, Rodica D. Catana

**Affiliations:** 1 CICTE, Department of Life Science and Agriculture, Universidad de Las Fuerzas Armadas—ESPE, Av. General Rumiñahui s/n y, Sangolqui 171103, Ecuador; 2 Department of Life Science and Agriculture, Universidad de Las Fuerzas Armadas—ESPE, Av. General Rumiñahui s/n y, Sangolqui 171103, Ecuador; 3 IASA 1, Department of Life Science and Agriculture, Universidad de las Fuerzas Armadas—ESPE, Av. General Rumiñahui s/n y Ambato, Sangolquí 171103, Ecuador; 4 Institute of Biology Bucharest of Romanian Academy, 296 Splaiul Independentei, 060031 Bucharest, Romania

**Keywords:** *Moringa oleifera* Lam., toxicity of nutrients, bioactive compounds, antioxidant activity, abiotic components

## Abstract

*Moringa oleifera* Lam. contains numerous essential constituents found in all plant parts (leaves, pods, and seeds). From all its edible parts, the leaf represents an effective remedy with high potential for medicinal applications. Ecuador is part of the new promising cultivation areas for *Moringa,* and therefore our study is emphasized to determine the influence of soil nutrition, toxicity (excess), and deficiency, from three main areas of this country, correlated with its climatic characteristics, on the mineral components, bioactive compounds’ synthesis, and antioxidant capacity of *Moringa*. Different analyses were performed in soil and especially leaf samples for phytochemical content, antioxidant activity, calcium, protein, and vitamin C determination to identify the relationship between soil nutrients, abiotic conditions, and the therapeutic potential of this species cultivated in Ecuador. The obtained values using methods such as DPPH, FRAP, and ABTS showed a high antioxidant capacity of the leaves from the Coastal Ecuadorian region, related with total phenolic compounds’ content (through the Folin–Ciocalteu method) and flavonoids in samples, with results obtained under the positive influence of high soil nutrients such as Ca, Mg, Mn, and Fe. We can conclude that *M. oleifera* from the coastal area of Ecuador presents the right environmental and soil conditions to positively influence its mineral and phytochemical content, making it suitable for incorporation into foods and medicines to solve the nutritional and medical problems in Ecuador and worldwide.

## 1. Introduction

*M. oleifera* represents one of the most known species of the *Moringa* genus, native to India and cultivated across the world, a fast-growing, drought-resistant plant species, that tolerates a wide range of soil conditions. It was acclimatized to the tropics and subtropics, being known as horse reddish or ben tree [1]. Recently, research concerning the growing/acclimatization of this species in Europe was also reported [2].

*M. oleifera* is a very important plant species used in traditional phytomedicine due to its nutrients. Various phytochemicals can be found among all parts of the plant, which makes them all edible [3]. It is considered a highly nutritional food, providing vitamins (vitamin C, vitamin A), minerals (calcium, potassium, iron, zinc), protein, phytosterols, amino acids, and PUFAs [4]. Scientific research outlines the appreciation of *M. oleifera’s* uses for different therapeutic applications (antihyperglycemic, cardio-protective, antiparasitic, antibacterial, antifungal, antiviral, cytotoxic, antioxidant, anti-inflammatory, type 2 diabetes mellitus, osteoporosis, anemia, dyslipidemias), as described by González-Burgos et al. [5]. *Moringa* also has other uses, such as in water purification [6], fertilizing, and green manure [4] as a bio adsorbent, coagulant, biodiesel, and biopesticide [7]. There is no evidence of its effectiveness as a plant fertilizer [8].

Even though there are numerous drug treatments for various diseases, the request for medicinal plants has shown a higher demand in the last decade. Plant-based foods are healthier and more suitable for vegetarians and allergic persons [9]. In this way, the identification of plant species with high nutritional values that can solve important health problems will always be a priority. 

Some of the world’s biggest health problems, such as malnutrition and osteoporosis, may be positively influenced by using a diet based on *M. oleifera*. Malnutrition represents a restricted diet consisting of the consumption of the same type of food daily [10]. Good nutrition should consist of a varied diet (meat and vegetable foods). Recently (2020), at the global level, more than 45% of deaths in the case of children under 5 years are due to malnutrition [10]. In Ecuador, in 2020, according to the World Bank, child malnutrition was reported at 0.66667% (higher than in 2014) [11]. Osteoporosis, a major health problem, represents a deterioration of the bone, which increases the fracture risk [12]. By 2050, it is expected that the number of individuals aged > 65 years suffering from osteoporosis will reach 1.555 million [13]. *Moringa* is rich in calcium, which plays an integral role in bone structure, contraction of muscle, and neuronal signaling [14]. A recent review reported an average intake of calcium < 400 mg/day in Asia and South America (Ecuador) since it is difficult for low-income countries to obtain adequate calcium from food [15].

*M. oleifera* has been introduced as an alternative food to overcome malnutrition [16]. There are studies concerning the benefits of *Moringa* for increasing the weight of children with poor nutrition [17,18].

Another worrying health problem is cancer, accounting for ~ 13% of deaths worldwide. Chemotherapy is the most frequent treatment of cancer, associated with adverse reactions [19]. Antioxidants may be used as adjuvants in chemotherapy due to their capacity to react and eliminate oxidizing free radicals. At present, 13–87% of patients with cancer are using antioxidant supplements [20]. The data obtained in different studies suggest that *M. oleifera* leaves’ extracts have potent antioxidant activity (comparable with that of the reference antioxidants), preventing the oxidative damage of major biomolecules, affording significant protection against oxidative damage [21]. 

Ecuador (South America) is located between the Pacific Ocean and the Amazon basin. The Andean Mountains cross the country from north to south, being a substantial topographic barrier [22]. In the Coastal region, the lithological composition is dominated by tertiary structures [23], and excellent agricultural soils (good granular structure with a high organic matter content and good drainage). The soil texture in the Andean region is of the loam type (soil of great agricultural productivity due to its relatively loose texture) [24]. In the Amazon region, the soils are taxonomically classified as belonging to the Inceptisol order, the large Tropaquept group. Brown silty clay loam soils are moderately deep, have moderate drainage, acidic pH (4.5–5.5), very high toxic aluminum, and very low fertility [25].

The phytochemical content varies by many factors, such as cultivars, soil types, toxicity and deficiency of soil nutrients, precipitation levels, light intensity, humidity, etc. [26]. In the case of *Moringa*, it was shown that high antioxidant activity varies between cultivars from different geographical regions, and the differences in phenolic content may suggest different adaptation abilities to face the various environments [23]. 

Considering the medicinal properties of *M. oleifera* and its widespread cultivation in different areas of Ecuador, it is important to evaluate, as in this study, the influence of the abiotic components such as soil nutrients (toxicity (excess) and deficiency) and ambient conditions characteristic for three areas, Andean, Amazonian, and Coastal regions, on the synthesis of constituents responsible for the biological effect and on its components with nutritional properties. In this view, the objective of the present study was to test the variability of the antioxidant potential and biochemical and nutritional constituents of *Moringa* under the influence of soil macro- and micro-nutrient toxicity (excess levels) and deficiency, under local climatic conditions characteristic of three different Ecuadorian regions.

## 2. Materials and Methods

### 2.1. Sample Collection 

#### 2.1.1. Sample Collection for Soil Analysis

For the soil analysis, a zig-zag sampling was carried out, trying to cover the entire lot to be studied belonging to elected Provinces from the three Ecuadorian areas. For this purpose, 20 subsamples, representing 10 cores collected from a 30 cm depth, were taken for each lot with a shovel, and then all the samples were mixed and transported in a cooler, in plastic bags, to the laboratory for further soil analysis using the standard procedures of the Agricultural Research and Education Center (AREC), Belle Glade. The pH of the soil was carried out in a 1:2.5 dilution (1 part soil in 2.5 parts deionized water).

**Figure 1:**
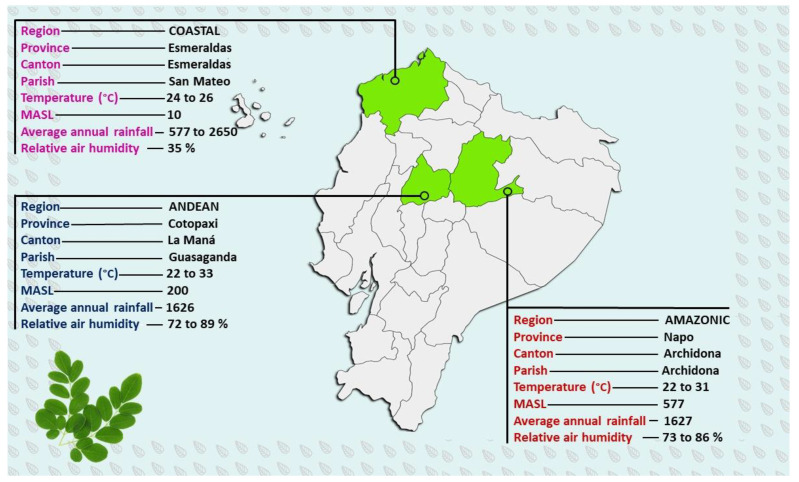
Ecuadorian *Moringa oleifera* growing sites. MASL: meters above sea level.

Plant Material was represented by the fresh young leaves collected from middle *M. oleifera* branches. The samples were selected from three Ecuadorian growing regions: Andean, Coastal, and Amazonian, chosen based on different altitudes and representative climatic characteristics, respectively (Figure 1).

#### 2.1.2. Sample Collection for Foliar Analysis

For the foliar analysis, *Moringa* leaves were collected from three different regions of Ecuador: the Coastal region, in the province of Esmeraldas at 10 MASL (meters above sea level), with a mollisol-type soil, the Andean region, at 200 MASL (Cotopaxi province, with a selected area with the lowest altitude in this region), with an inceptisol-type soil, and from the Amazonian region, represented by Napo province at 577 MASL, with an inceptisol-type soil. The study was carried out in already established plantations, with 2 × 2 m planting distances and an average age of four years for all studied areas. The collected leaves were taken from the middle third of the trees, out of a total of 60 fully developed compound leaves, collected from 15 trees and transported in the right conditions (paper bags) for analysis. The sampling date was carried out in August 2021. In the laboratory, the leaves were washed (with neutral soap and deionized double-distilled water) and dried for 24 h at 80 °C. After drying, the total N (by micro-Kjeldahl) was analyzed from the grounded material. The elements K, Ca, Mg, Fe, Zn, and Cu were analyzed by atomic absorption spectrophotometry, P was identified by a colorimetric method using vanadate molybdate reaction, Bo through a turbidimetric method, and S through photometry. The standards developed by Marrocos et al. [27] were used to compare the results concerning the macronutrients and micronutrients.

To assess the effect of regional variations in the phenolic and flavonoid content of *M. oleifera*, the samples were collected in the same conditions (time—in the morning hours, and maturity—plants older than three months) from three Ecuadorian regions characterized by different climatic and nutrient characteristics. 

#### 2.1.3. Extracts’ Preparation

The shade-dried leaves were macerated with methanol for 24 h at room temperature. A rotary evaporator was used for evaporation of the liquid, and the extracts were freeze-dried. The process was repeated 3 times. 

### 2.2. Phytochemical Analysis

#### 2.2.1. Quantification of Total Phenolic Content (TPC)

The method used for TPC quantification was based on the methodology described by Madaan et al. [28], with modifications, consisting of the Folin–Ciocalteau colorimetric method [29]: A portion of the *Moringa* extract (diluted to 5 mL of Milli-Q water) was added to the 1.5 mL Folin–Ciocalteau reagent, and kept for 5 min for reaction at room temperature (25 °C). After that, 2 mL of a 100 g/L solution of Na_2_CO_3_ was added. The absorbance was measured at 760 nm in a spectrophotometer against a blank (without extract) after 30 min. A calibration curve with standard solutions within a range of 50–250 mg/L was obtained using gallic acid, with a correlation coefficient of 0.9968 (DL = 0.7643 mg/L, QL = 2.5477 mg/L (5.9701%), n = 6, *p* = 3.77 × 10^6^). The results were expressed as mg GAE/100 g DW. 

#### 2.2.2. Quantification of Total Flavonoid Content (TFC) 

The method was realized using the Dowd method [30]. A volume of 1 mL of *Moringa* extract solution (25–200 µg/mL) was added to 0.3 mL of 10% (*v*/*v*) AlCl_3_ solution in methanol, 0.2 mL (1 M) of potassium acetate, and 5.6 mL of distilled water, and left to rest for 10 min at room temperature. The absorbance was read at 430 nm. A range of 1–100 mg/L of quercetin (QE) was used as a standard for the calibration curve, with a coefficient of 0.9991 (DL = 0.4752 mg/L, QL = 1.5843 mg/L (11.5003%), y = 0.0167x + 0.0245, n = 7, *p* = 8.79 × 10^9^). The TFC results were expressed as mg quercetin equivalents/100 g *Moringa* extract dry weight.

### 2.3. Antioxidant Analysis

#### 2.3.1. FRAP Assay

The method used for the FRAP assay was described by Benzie et al. [31]. Acetate buffer (300 mM, pH 3.6), a solution of 10 mM of TPTZ in 40 mM of HCl, and 20 mM of FeCl_3_ at 10:1:1 (*v*/*v*/*v*) were mixed to obtain the FRAP reagent. The sample was incubated with 2 mL of the FRAP solution at 37 °C. The FRAP solution was prepared the same day by mixing the following reagents: acetate buffer (25 mL), TPTZ solution (5 mL), and FeCl_3_·6H_2_O solution (10 mL), for 30 min in the dark. The absorbance was read at 593 nm. Six standard solutions of ferric sulfate (Fe_2_SO_3_) in the range of 0.10–1.00 mmol/L were used for the calibration curve (y = 1.5431x + 0.0004, R^2^ = 0.9964, n = 7, *p* = 3.29 × 10^7^, DL = 0.005939 mmol/L, QL = 0.01979 mmol/L (0.3740%)).

#### 2.3.2. ABTS Assay

The method described by Loizzo et al. [32] was used for the determination of ABTS free radical scavenging activity by mixing ABTS (2 mM) and potassium persulfate (70 mM). The mix was kept at room temperature in the dark overnight. To obtain an absorbance of 0.700 ± 0.005 at 734 nm, the ABTS solution was diluted with 80% methanol, and 100 μL of appropriately diluted samples was added to 2 mL of ABTS solution. The absorbance was recorded after 1 min of incubation at room temperature at 734 nm. TROLOX standard solutions in the range of 10.0–150.0 mg/L were used for the standard curve, with the equation y = 0.5426x − 0.0069 (R^2^ = 0.9903, n = 8, *p* = 0.00102, DL = 0.01796 mg/L, QL = 0.05989 mg/L (0.3250%)).

#### 2.3.3. DPPH Assay 

The method presented by Simirgiotis et al. [33] was used to determine the DPPH^•^ radical scavenging activity, consisting in 50 µL of extract added with 2 mL of fresh 0.1 mM solution of DPPH in methanol, kept at room temperature in the dark. The absorbance of the mix was measured after 30 min at 517 nm. The DPPH scavenging ability (%) was calculated with the formula (A_control_ − A _sample_/A _control_) × 100. The IC_50_ (concentration of sample required to scavenge 50% of the DPPH free radicals) values were calculated. A low IC_50_ value means a powerful antioxidant activity. Then, 10.0–100.0 mg/L of TROLOX standard was used for the calibration curve expressed by the equation y = 0.9081x + 0.08 (R^2^ = 0.9542, n = 7, *p* = 0.000151, DL = 0.00809 mg/L, QL = 0.02698 mg/L (0.2970%)).

Calcium determination was realized by atomic absorption spectrophotometry (AAS) according to the method presented by Allen [34], whereby 0.1 g of sample (dry leaves) was ashed at 525 °C for 8 h and dissolved with 0.1% HCl and 0.5% lanthanum oxide (La_2_O_3_).

Protein content was estimated by the Kjeldahl method [35] at the nitrogen conversion factor of 6.25, consisting in a mixture of 2 g of dry sample, 7 g of the catalytic mixture, and 15 mL of 96% sulfuric acid. Tubes were placed in a heating block (450 °C) for 1 h. A 4 min distillation was carried out after cooling with the addition of 32% NaOH until the neutralization of the mixture. The distillation product had 0.1 N HCl. The total protein content (%) was calculated by the formula: the total volume (mL) used to neutralize the mix × 0.1 × 1.4 × 5.7/W the weight of the sample

Vitamin C content (expressed as mg of ascorbic acid/100 g of fresh weight) was determined using the procedure reported by Xiao et al. [36] based on 2.6-dichloroindophenyl, and the content was measured with the titrimetric method.

### 2.4. Statistical Analysis 

For the data analysis of soil and leaf samples, a comparison was made with the works of different authors [37,38,39,40] who have worked with *M. oleifera*. For the soils, the sufficiency interpretation of the INIAP laboratory was used, and for the determination of the relationships between the cations Ca^2+^, K^+^, and Mg^2+^, a table of general relationships that predict the possible deficiency of the element in the plant was used. To analyze the phytochemical and antioxidant activity, three replications were used, and the results are expressed as means ± SD (standard deviation). Spearman’s correlations were used to determine the relationship between secondary metabolite content and antioxidant capacities. The Kruskal–Wallis test with post-hoc Dun multiple pairwise comparisons was applied to emphasize the differences, taking into account the geographical origin of the samples of secondary metabolites and their antioxidant capacities. The Kruskal–Wallis test is a non-parametric method alternative of one-way ANOVA. The statistical analysis was performed with XLSTAT pro (2013) [41]. Principal component analysis was used to compare the samples using values of secondary metabolites and antioxidant activity.

## 3. Results

### 3.1. Soil and Leaf Analysis

The results concerning the nutrients from the soil and foliar samples are presented in Table 1 and Table 2.

The Coastal region presented a higher nitrogen content (4.72%) than the other regions (*p* = 0.00381). The average of the Ecuadorian regions was 4.60, in line with the values identified by Moyo et al. [37] and higher than those of Valdez-Solana et al. [38] (Table 1). For phosphorus, the results were very similar in the three regions, with an average of 0.32%, close to those of Moyo et al. [37] and Witt and Aslam et al. [39,40]. The highest potassium content (2.44%) was found in the Amazonian followed by the Coastal and the Andean regions, respectively. The average of the three zones under study was 2.31%, and these values are directly related to the potassium levels in the soil (Table 2). 

The soil cation ratios K/Mg, (Ca+Mg)/K, and Ca/K for the Coastal and Andean regions indicated a potassium deficiency. In the Amazonian region, the ratio between (Ca+Mg) and K is the only one that shows deficiency for this nutrient. For sulfur, the average in the three areas under study was 0.57%, with the Coastal and Amazonian regions having the highest percentages (Table 3). 

Calcium is an element that is closely related to the amount of potassium, magnesium, and other cations. In this study, the average for the three studied areas was 2.23%, with the Coastal region having the highest content, followed by the other two regions. This average was lower compared with the results of Moyo et al. [37], Valdez-Solana et al. [38], and Aslam et al. [39], and slightly higher than those of Witt [40].

In the case of magnesium, the average for the three study regions was 0.26%, which is lower than that of the other authors, except Aslam et al. [39]. The Coastal and Amazonian regions have lower Mg content than the Andean region. This can be attributed to the cationic Ca/Mg ratios, which show a probable magnesium deficiency in the soil in the three regions.

In the foliar analysis, the iron value was higher in the Andean region compared to the Coastal and Amazonian regions. The average of the three regions under study was 108.80 mg/kg, a low level compared to the data of Moyo et al., Valdez-Solana et al., and Witt and Aslam et al. [37,38,39,40]. 

Leaf zinc contents on average were low compared to those of Moyo et al. and Witt and Aslam et al. [37,38,39,40], and higher than those of Valdez-Solana et al. [38]. However, in the soil analysis, zinc cation was found at a high level in the Coastal region and was optimal in the Amazonian region. The high contents of calcium and iron cations inhibit the absorption of zinc cation in the plant.

The copper content in leaves was similar, and the average of the three regions was close to the ranges of Moyo et al. [37], Witt, and Aslam et al. [39,40], and lower than those of Valdez-Solana et al. [38]. This also coincides with the soil values.

### 3.2. Phytochemical Analysis

The total phenolic and flavonoid contents were determined, and the Kruskal–Wallis test showed significant geographical differences in TPC (*p* = 0.027), with the Dunn post-hoc test (*p* = 0.007), between the Coastal and Amazonian regions. The same results were found for TFC (*p* = 0.027), with differences between the Coastal and Amazonian regions (Dunn post-hoc test, *p* = 0.007). The highest TPC values were established in the sample from the Coastal region (152.6947 ± 4.0104 mg/100 g DW) and the lowest (43.6796 ± 4.0477 mg/100 g DW) in the Amazonian region. The same trend was observed for TFC, with the highest content being in the Coastal region (74.133 ± 4.618 mg QE/100 g DW) and the lowest in the Amazonian region (20.7 ± 0.917 mg QE/100 g DW) (Table 4).

### 3.3. Antioxidant Analysis

Table 5 shows the antioxidant activity of *Moringa* samples collected from the three Ecuadorian regions. Significant differences between Coastal and Amazonian regions were observed. All three methods used for antioxidant capacity (FRAP, ABTS, and DPPH assays) showed the same trend, with the highest antioxidant capacity being found in the samples collected from the Coastal followed by the Andean and Amazonian regions (Table 5). On the other hand, in the samples from the Coastal region, it was noticed that TFC was significantly correlated with FRAP activity (*p* < 0.0001) and DPPH (*p* < 0.0001). In the Andean region, TFC was correlated with DPPH (*p* < 0.0001), and in the Amazonian region with FRAP (*p* < 0.0001). Concerning the TPC, no significant correlation was observed between TPC and antioxidant activity in the studied regions.

### 3.4. Calcium and Protein Content Analysis in Moringa Leaves

The calcium content of the samples varied from 1.78% ± 0.05% in the Andean region to 2.50% ± 0.04% in the Coastal region (Table 4). Significant differences (Kruskal–Wallis test, *p* = 0.027) were observed between Amazonian and Coastal regions (Dunn post-hoc test, *p* = 0.007) concerning protein content (%). Leaves from the Coastal region were characterized by the highest protein content, correlated with the highest calcium content. Additionally, leaves from the Coastal region were characterized by highest amount of vitamin C/100 g leaves, followed by the Andean and Amazonian regions (Table 6).

### 3.5. Multivariate Analysis

**Figure 2:**
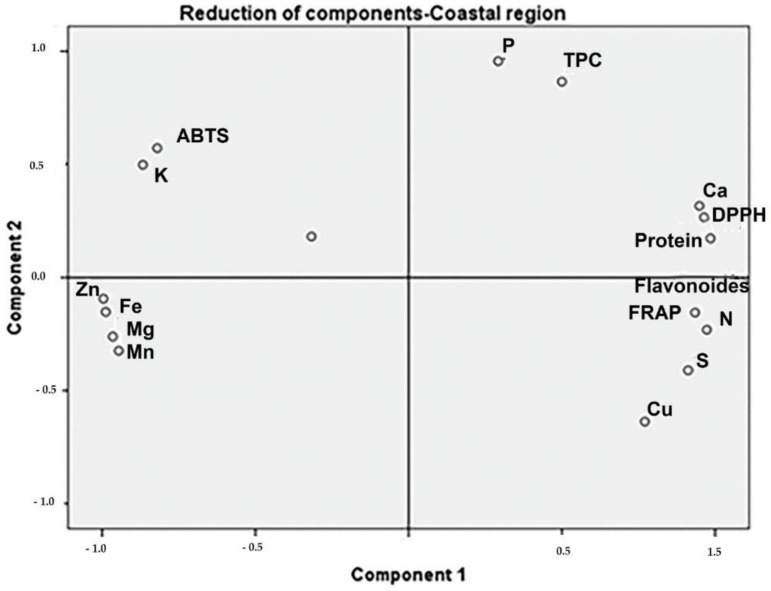
The phytochemical and biological composition of *M. oleifera* from the Coastal region. Loading plot of PC1 versus PC2, showing the link between DPPH with TPC, calcium, and protein. Legend: TPC—total phenolic content, TFC—total flavonoid content, FRAP—ferric-reducing antioxidant power, ABTS—free radical scavenging activity, DPPH—free radical scavenging ability by the use of a stable DPPH^•^ radical.

**Figure 3:**
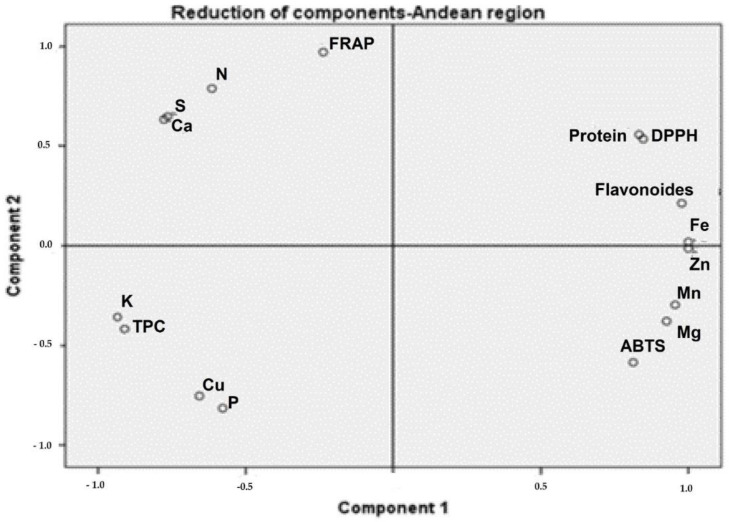
The phytochemical and biological composition of *M. oleifera* from the Andean region. Loading plot of PC1 versus PC2, showing the link between DPPH with TFC, Fe, and protein. Legend: TPC—total phenolic content, TFC—total flavonoid content, FRAP—ferric-reducing antioxidant power, ABTS—free radical scavenging activity, DPPH—free radical scavenging ability by the use of a stable DPPH^•^ radical.

**Figure 4:**
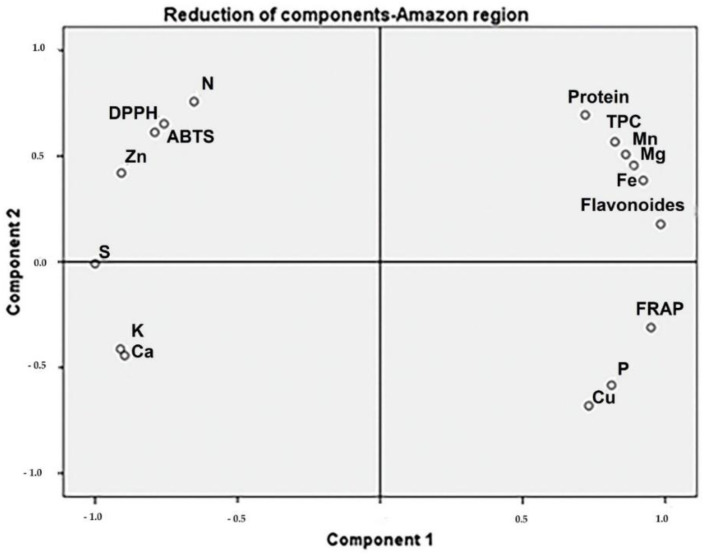
The phytochemical and biological composition of *M. oleifera* from the Amazonian region. Loading plot of PC1 versus PC2, showing the influence of Mn, MG, Fe, TFC, and TPC. Legend: TPC—total phenolic content, TFC—total flavonoid content, FRAP—ferric-reducing antioxidant power, ABTS–free radical scavenging activity, DPPH—free-radical-scavenging ability by the use of a stable DPPH^•^ radical.

*Moringa* samples collected in the Coastal region showed positive PC1 scores, associated with TPC, calcium, protein, and DPPH (Figure 2). The samples collected in the Andean region presented high protein and TFC associated with DPPH (Figure 3). The protein content of samples from the Amazonian region was correlated with Mn, Mg, and Fe levels. Additionally, TPC and TFC were influenced by these macro-elements (Figure 4). 

## 4. Discussion

The nutritive compounds as well as secondary metabolites depend on different factors (meteorological, geographical, toxicity (excess), and deficiency of macro- and micro-nutrients in soil) [38]. In general, the nutrient quality of the plants is the same, but is influenced by geographical location and environmental conditions [37]. For this reason, for each region analyzed in our investigation there are different nutrients that influence the accumulation of metabolites, protein, and vitamins in the *Moringa* leaves. In the case of the Coastal region, the metabolites, as phenolic compounds (that also give the antioxidant property of *Moringa*), are influenced positively by Ca in leaves (calcium uptake from the soil). The Coastal region, based on its climatic characteristics, can provide an efficient absorption of this element due to the help of transpiration (calcium rises to the level of the leaves when the plant transpires). This region is the warmest among all the regions described, and the collection was performed in August when the low humidity made the plant sweat so it could accumulate calcium in the leaves. In the other areas of Ecuador where the climatic conditions do not support the plant’s transpiration, the accumulation of calcium in leaves is not supported. Murtadha et al. [42] showed that the absorption of calcium may be slowed down by high temperatures and relative humidity. Ca^2+^ has a role in signaling, helping in the regulation of genes for biosynthesis of polyphenols [43]. For the Amazonian region, the protein and secondary metabolite contents in the *Moringa* leaves were influenced by nutrients such as Mn, Mg, and Fe. These elements were proven to affect plant defense metabolites, having interactive effects on the development of phenols and flavonoids [44], which is a strong reason for secondary metabolites’ accumulation in the leaves of *Moringa* in the presence of these nutrients. The soil nutrients Mn, Mg, and Fe can be found in similar concentrations in Coastal and Amazonian regions and can positively influence the accumulation of desirable compounds, such as phenolics. We can observe a difference between the metabolites’ content in these two regions due to the environmental factors characteristic of each area, responsible for fluctuations in *Moringa’s* nutritive qualities and secondary metabolites’ accumulation due to different nutrient uptake or nutrient translocation in the plant. The abiotic conditions of the Amazonian region are characterized by an increased relative humidity (RH) and high moisture content in the atmosphere, reducing the nutrient translocation through reduction of the xylem volume. The Coastal area, in comparison, presents a low RH and the atmosphere contains more moisture, and in this way the driving force for transpiration is increased, improving the translocation of minerals in plants, especially the nutrients responsible for metabolite accumulation. 

Our results showed that the average values of nutrients from soil samples collected from Ecuadorian regions were similar to those in other works. The average nitrogen and phosphorus contents were closer to the values identified by Moyo et al. [37]. The nitrogen content from the Coastal region is directly related to the protein content. Phosphorus content was very similar in the three regions.

Despite the high (toxic) iron content in the soil from the Coastal region, this element is affected in its absorption by the high contents of calcium, manganese, and zinc present in the soil. Additionally, zinc is found at a high level in the Coastal region and is optimal in the Amazonian region, and the high contents of calcium and iron inhibit the absorption of zinc in the plant.

The so-called miracle tree was already widely used in ancient times for its nutritious and healthy properties due to its diverse pharmacological activity [4]. The present findings provide an insight into the polyphenol and flavonoid contents in *M. oleifera* leaves depending on the cultivation area. Previous studies reported that *M. oleifera* extract is rich in phenolic and flavonoid compounds. The quercetin, kaempferol, and rutin described in *M. oleifera* are associated with environmental and growing factors [45]. Leaves from the Coastal region were abundant in phenols and flavonoid contents. The amounts of phenols found in the leaves collected from this area were similar to those collected in Mexico [46], but lower than those from Southwestern Algeria and Haiti (2813 ± 51 mg GAE/100 g DW) [47]. The case of flavonoid content followed the same trend as phenolic content, with the highest flavonoid concentration expressed in the leaves collected from the Coastal region, and the lowest reported in the Amazonian region. Our study confirmed the importance of growing regions on the synthesis of TPC and TFC, and was compared with other results, where these constituents from lower altitudes were higher than in other regions [46]. In previous reports, flavonoids were represented by quercetin and kaempferol derivates [47].

*M. oleifera* demonstrates, in all the scientific reports including ours, that it is a plant booming in polyphenols, with strong antioxidant properties, and may decrease the oxidative damage of the tissues by scavenging free radicals [4]. The *Moringa* genus is characterized by high antioxidant activity caused by its high content of bioactive polyphenols, a fact also demonstrated in our experiment that could be visualized on the leaves, with the highest polyphenolic content in the Coastal region, which also expressed the highest antioxidant capacity compared to the leaves collected from the other Ecuadorian regions. Three different methods (DPPH, ABTS, and FRAP) were used to establish the antioxidant potential of *M. oleifera* leaves from different geographical Ecuadorian regions. The results of our study indicate a descending order of antioxidant activity from leaves collected in the Coastal region < Andean region < Amazonian region, with the highest values of antioxidant activity being similar to those in some other investigations [48].

The *Moringa* leaves collected from the three Ecuadorian regions contained relatively high amounts of the examined minerals. Our results showed that the highest value of calcium was in leaves collected from the Coastal region, which was found to be a good source of calcium (2.50%), with the obtained values being similar to others reported in a bibliography about *Moringa* sp. leaves’ calcium content [48]. A diet containing vitamin C helps to maintain the human body’s health, being an essential micronutrient for normal functions, supplied by fruits and vegetables. Vitamin C is a potent antioxidant that can eradicate different reactive oxygen species, has a role in stress resistance, keeps membrane-bound antioxidant α-tocopherol in a reduced state, and is a substrate for oxalate and tartrate biosynthesis [49]. In our study, the highest vitamin C concentration could be found in the leaves from the Coastal region, which also coincides with a high antioxidant activity, demonstrating the contribution of vitamin C to this desirable plant property. Our results are in line with earlier findings on reported *Moringa oleifera* vitamin C content [49]. Proteins are indispensable for the normal functions of the body. The implication of proteins in the growth and maintenance of tissues is well-known. Additionally, proteins are implicated in functions of the body as chemical messengers (such as hormones), aiding the communication between cells, tissues, and organs, providing stiffness and rigidity. Proteins have a vital role in regulating acid and base concentrations in blood and other fluids, helping to form the immunoglobulins, or antibodies, and in this way helping the organism to fight infections [50]. *Moringa* is recognized to contain proteins, and from this point of view, its inclusion in humans’ and animals’ diets could represent a curative and therapeutic therapy. The trend is different than in the case of secondary metabolites because although the highest protein content was found in leaves from the same Coastal region, in second place is the Amazonian region, while the Andean region was second highest for secondary metabolites. A possible explanation could be that the highest N content was found in the soils from the Amazonian region, compared to the deficit of this nutrient in the Andean region, as revealed by our experiment. The importance of N for the proteins consists of the use of nitrogen translocated from the soil in both the xylem and phloem for plant protein synthesis [51].

Even if the majority of soil nutrients are in a higher content in the Amazonian region, the *Moringa* leaves with high biochemical, nutritional, and therapeutic properties were found in the Coastal region. These findings may be explained by the interaction between climatic characteristics (temperature, humidity, etc.) and soil type. 

Through this research, we support the traditional idea that *Moringa* represents a good source for the human and animal diet, providing useful constituents such as vitamins, proteins, and minerals. The effects of abiotic components from three Ecuadorian climatic regions on the biochemical and nutritional properties of *Moringa oleifera* were studied. Our research represents a general picture of environmental factors and nutrients of three Ecuadorian regions (Amazonian, Andean, and Coastal), responsible for fluctuations in the nutrient and metabolomics composition of *M. oleifera.* There were variations observed in phytochemical content, antioxidant activity, calcium, protein, and vitamin C content in samples from different regions due to abiotic factors such as relative humidity, which influence the soil nutrients’ uptake and translation into the plant, with nutrients such as Mg, Mn, and Fe being responsible for the accumulation of metabolites of interest, such as phenolics. Our results are in accordance with those found in *Aloe vera,* where Mg, Fe, and Mn levels are directly corelated with pharmaceutical compounds represented by TPC and TFC [52]. Additionally, the authors of [53] underline that the application of compost increases the TPC and TFC.

The highest biochemical and nutritional properties were found in *Moringa* leaves cultivated in the Coastal region, due to the low relative humidity that triggered the high transpiration rate and implicit minerals’ uptake, as well as the low altitude that was demonstrated to have a positive influence on secondary metabolites’ accumulation in *Moringa*. Such information has high importance in the context of developing new areas for the cultivation of plants such as *Moringa* with a high therapeutic potential. The investigation outlined the importance of the environmental and nutritional conditions (toxicity (excess) and deficiency) to obtain high-quality and high-quantity bioactive compounds, which can increase the nutritive qualities of *M. oleifera.*

## Figures and Tables

**Table 1:** Abiotic components from the studied regions in agreement with soil analysis.

Element/Unit	Coastal Region/Diagnosis	Andean Region/Diagnosis	Amazonian Region/Diagnosis
N (ppm)	31 ± 0.756/Medium	8 ± 0.266/Low	58 ± 0.337/Medium
P (ppm)	12 ± 0.684/Medium	8 ± 0.0603/Low	12 ± 0.724/Medium
K (meq/100 g)	0.06 ± 0.0025/Low	0.09 ± 0.005/Low	0.84 ± 0.032/High
S (ppm)	No determination/No determination	No determination/No determination	No determination/No determination
Ca (meq/100 g)	3.65 ± 0.056/High	7.65 ± 0.695/High	15.53 ± 0.557/High
Mg (meq/100 g)	0.57 ± 0.043/Medium	0.55 ± 0.0642/Medium	1.92 ± 0.080/High
Fe (ppm)	325.602 ± 8.409/High	No determination/No determination	230.7 ± 22.03/High
Mn (ppm)	16.238 ± 1.856/High	No determination/No determination	18.25 ± 0.854/High
Zn (ppm)	3.869 ± 0.269/High	No determination/No determination	3.91 ± 0.229/High
Cu (ppm)	7.08 ± 0.703/High	No determination/No determination	5.75 ± 1.071/High
B (ppm)	<0.5 ± 0.038/No determination	No determination/No determination	0.15 ± 0.038/Low

**Table 2:** Abiotic components from the studied regions in agreement with leaf analysis.

	Ecuadorian Regions				Comparison			
Element/ Unit	Coastal	Andean	Amazonian	Average of the 3 Regions	Moyo et al. * [37]	Valdez-Solana et al. * [38]	Witt [39]	Aslam et al. [40]
**N (%)**	4.72 ± 0.121	4.39 ± 0.04	4.69 ± 0.045	4.60 ± 0.182	4.8	1.78	ND	ND
**P (%)**	0.32 ± 0.021	0.32 ± 0.009	0.33 ± 0.004	0.32 ± 0.006	0.3	ND	ND	0.13
**K (%)**	2.31 ± 0.045	2.17 ± 0.036	2.44 ± 0.04	2.31 ± 0.135	1.5	1.83	1.46	2.17
**S (%)**	0.62 ± 0.043	0.5 ± 0.018	0.6 ± 0.026	0.57 ± 0.064	0.63	ND	ND	ND
**Ca (%)**	2.50 ± 0.046	1.78 ± 0.026	2.40 ± 0.02	2.23 ± 0.39	3.65	2.32	1.9	2.27
**Mg (%)**	0.2 ± 0.026	0.37 ± 0.01	0.2 ± 0.017	0.26 ± 0.098	0.5	0.33	0.47	0.1
**Fe (mg/kg)**	99.98± 4.105	129.46 ± 5.066	96.96 ± 1.093	108.80 ± 17.955	490	130	325	391.67
**Mn (mg/kg)**	34.99 ± 1.22	131.96 ± 1.505	35.49 ± 0.429	67.48 ± 55.842	86.8	ND	ND	95.8
**Zn (mg/kg)**	18.5 ± 0.917	20.49 ± 1.767	19.49 ± 1.64	19.49 ± 0.995	31.03	13	24	21.73
**Cu (mg/kg)**	9.5 ± 0.398	9 ± 0.0174	9 ± 0.217	9.17 ± 0.289	8.25	14.4	9	9.33
**B (mg/kg)**	ND	ND	ND	ND	49.93	ND	ND	ND

Legend: * Divided by 6.25 to determine the N. ND: No determination.

**Table 3:** The cationic relationships in soil between the three analyzed regions, optimum and deficiency ranges.

	Coastal Region	Analysis	Andean Region	Analysis	Amazonian Region	Analysis
**K/Mg**	0.11 ± 0.006	Def K	0.16 ± 0.013	Def K	0.44 ± 0.031	Def Mg
**Ca/Mg**	6.40 ± 0.388	Def Mg	13.91 ± 0.783	Def Mg	8.09 ± 0.376	Def Mg
**(Ca+Mg)/K**	70.33 ± 2.269	Def K	91.11 ± 3.498	Def K	20.77 ± 0.754	optimum
**Ca/K**	60.83 ± 2.144	Def K	85.00 ± 3.096	Def K	18.49 ± 0.613	optimum

**Table 4:** Total phenolic and flavonoid contents in *Moringa* leaves from three Ecuadorian regions.

	TPC (mg GAE/100 g DW)	TFC (mg QE/100 g DW)
**Coastal region**	152.6947 ± 4.01	74.133 ± 4.618
**Andean region**	79.2014 ± 4.49	37.4667 ± 2.201
**Amazonian region**	43.6796 ± 4.047	20.7 ± 0.917

Legend: TPC—total phenolic content, TFC—total flavonoid content.

**Table 5:** Antioxidant activities in *Moringa* leaves from three Ecuadorian regions.

	FRAP (mg Ferric Sulfate/100 g DW)	ABTS (mg TEAC/100 g DW)	DPPH (mg TEAC/100 g DW)
**Coastal region**	821.4012 ± 19.883	41.866 ± 1.321	29.737 ± 1.303
**Andean region**	575.0679 ± 0.993	32.247 ± 3.034	25.523 ± 3.034
**Amazonian region**	409.7345 ± 5.138	17.588 ± 2.302	19.496 ± 2.302

Legend: FRAP—Ferric-reducing antioxidant power, ABTS—free radical scavenging activity, DPPH—free radical scavenging ability by the use of a stable DPPH^•^ radical.

**Table 6:** Calcium and protein content in *Moringa* leaves from different Ecuadorian regions.

	Calcium Content %	Protein Content %	Vitamin C (mg/100 g Leaves)
**Coastal region**	2.50% ± 0.04%	35.48% ± 0.86 %	156.85 ± 1.652
**Andean region**	1.78% ± 0.05%	23.13% ± 0.20%	113.28 ± 0.725
**Amazonian region**	2.40% ± 0.01%	32.79% ± 0.95%	86.79 ± 1.285

## Data Availability

Not applicable.
